# Seabuckthorn Paste Protects Lipopolysaccharide-Induced Acute Lung Injury in Mice through Attenuation of Oxidative Stress

**DOI:** 10.1155/2017/4130967

**Published:** 2017-08-16

**Authors:** Leilei Du, Xiaoxin Hu, Chu Chen, Tingting Kuang, Hengfu Yin, Li Wan

**Affiliations:** ^1^College of Ethnomedicine, Chengdu University of Traditional Chinese Medicine, Chengdu, China; ^2^College of Pharmacy, Chengdu University of Traditional Chinese Medicine, Chengdu, China; ^3^Sichuan Academy of Chinese Medicine Sciences, Chengdu, China

## Abstract

Oxidative stress is one of the major mechanisms implicated in endotoxin-induced acute lung injury. Seabuckthorn paste (SP), a traditional Tibetan medicine with high content of polyphenols and remarkable antioxidant activity, is commonly used in treating pulmonary diseases. In the present study, the protective effects and possible underlying mechanisms of SP on lipopolysaccharide- (LPS-) induced acute lung injury in mice were investigated. It was found that body weight loss, lung tissue microstructure lesions, transvascular leakage increase, malondialdehyde augmentation, and the reduction of superoxide dismutase and glutathione peroxidase levels caused by LPS challenge were all consistently relieved by SP treatment in a dose-dependent manner. Moreover, accumulation of nuclear factor erythroid 2-related factor 2 (Nrf2) in lung nuclei caused by SP treatment was observed. Our study demonstrated that SP can provide significant protection against LPS-induced acute lung injury through maintaining redox homeostasis, and its mechanism involves Nrf2 nuclear translocation and activation.

## 1. Introduction

Acute lung injury (ALI) is a multifactorial process which occurs due to various environmental triggers that include those caused by direct and indirect lung injury. With persistent high morbidity and mortality owing to complicated pathogenesis and a pathological course, ALI has been drawing increasing attention as a critical disease in clinics. According to previous reports, ALI, induced either by hypoxic stress or by chemical irritation of endotoxin, is typically accompanied with alteration of redox homeostasis and oxidative damage to lipids, proteins, and DNA. Hypoxic stress significantly enhanced the oxidative stress markers such as free radicals and malondialdehyde, and it is accompanied with decreased levels of antioxidants such as glutathione, glutathione peroxidase, and superoxide dismutase [1, 2]. In the pathogenesis of lung injury caused by lipopolysaccharide (LPS), free radicals have been verified as the final causative molecules with both *in vitro* and *in vivo* evidences [3]. Trials involving antioxidant supplementation revealed consistent results, among which alleviated damage to macromolecules induced by high altitude or endotoxin was observed [1–3].

Seabuckthorn (*Hippophae rhamnoides* L., Elaeagnaceae) has been used for a long history in Tibetan folk medicine. It is considered as a valuable herb in treating certain diseases including pulmonary conditions and in easing syndromes in unacclimatized persons on ascent to high altitude. Traditionally, seabuckthorn berries are collected and stewed in water before the extracted solution is filtered and condensed into a paste (seabuckthorn paste (SP)), which is a common preparation used in clinical practice. The edible berries of seabuckthorn contain a wild variety of oxidation-resistant compounds including relative high contents of vitamins and polyphenols [4] and can serve as a desirable natural antioxidant mixture. Based on these facts, we hypothesized that administration of seabuckthorn paste may enhance the antioxidant defense system and thus provide protection against ALI.

The transcription factor Nrf2 (nuclear factor erythroid 2-related factor 2) is a major regulator of the adaptive response to oxidative stress and orchestrates the expression of a large battery of cytoprotective genes such as antioxidants, phase II enzymes, and membrane transporters [5]. Under quiescent conditions, the transcription factor Nrf2 interacts with the actin-anchored protein Keap1, largely localized in the cytoplasm. This quenching interaction maintains low basal expression of Nrf2-regulated genes. However, upon recognition of chemical signals imparted by oxidative and electrophilic molecules, Nrf2 is released from Keap1, escapes proteasomal degradation, translocates to the nucleus, and transactivates the expression of several dozen cytoprotective genes that enhance cell survival [6].

Therefore, the present study was undertaken to investigate the effects of seabuckthorn paste in LPS-induced ALI in mice and to explore the possible mechanism in a perspective of redox homeostasis through its influence on the Nrf2 pathway.

## 2. Materials and Methods

### 2.1. Plant Material

Well-ripened seabuckthorn berries were collected from a natural growth site of a hilly region in the eastern margin of the Tibetan Plateau (Ma'erkang, Sichuan Province, China). Voucher specimens (number MZC-SJ-20160915-00~08) of the plant material are preserved in the herbarium of Chengdu University of Traditional Chinese Medicine after botanical identification.

### 2.2. Preparation of Seabuckthorn Paste

According to traditional process, 1 kg of seabuckthorn berries was boiled in 5 L water for 30 min and the supernatant was decanted, and the residue was reboiled in fresh water. The process was repeated three times for complete extraction. All the supernatants were pooled and filtered through a muslin cloth and condensed on a heater to obtain SP. The yield of SP from fresh berries was 13.88%, and the water content was 33.52%, calculated gravimetrically.

### 2.3. Antioxidant Activity *In Vitro*

Free radical scavenging activity was studied using 1,1-diphenyl-2-picrylhydrazyl (DPPH) while the total radical scavenging capacity was determined by the 2,2-azinobis-(3-ethylbenzothiazoline-6-sulfonic acid) (ABTS) assay, according to the procedures described by Debnath et al. [7]. Also, hydroxyl radical scavenging capacity was evaluated by the method described by Hazra et al. [8]. Trolox was used as standard, and the ability to scavenge radicals was expressed as IC_50_, which is defined as the concentration of the tested material required to cause a 50% decrease in initial DPPH/ABTS/hydroxyl radical concentration.

The contents of total polyphenols and total flavonoids in SP were determined by colorimetry according to the method described in previous reports [9, 10].

All measurements were performed in triplicate and reported as mean ± standard deviation (SD).

### 2.4. Animals

Six to eight-week-old male SPF KM mice weighing 20 ± 2 g were purchased from Dashuo Biotechnology Co Ltd. (Chengdu, China). The animals were maintained at 24 ± 0.5°C with food and water ad libitum. The experimental protocol is shown in Table 1. The study protocol was approved by the institute's animal ethical committee and conformed to the national guidelines on the use and care of laboratory animals.

After acclimatization for 2 days, the mice were randomly allocated into groups. The animals in the SP treatment groups received respective dose of SP, while the animals in the control and model groups received saline, once daily through intragastric route for seven consecutive days. On Day 8, the mice of all groups except for the control group were administered 10 mg/kg LPS (O55:B5) (Sigma, USA) intraperitoneally. The animals were sacrificed 10 h after LPS injection.

### 2.5. Body Weight Changes

The body weight of each mouse in each group was recorded before and 10 h after LPS injection, and body weight ratio was calculated by the formula: *R* = (Wa − Wb)/Wb, of which Wa and Wb represent body weight recorded 10 h after and immediately before LPS injection, respectively.

### 2.6. Bronchoalveolar Lavage Fluid (BALF) Analysis

A median sternotomy was performed for exposure of both lungs. The trachea was exposed and an intravenous infusion needle was inserted. The lungs were lavaged three times with 0.5 ml of ice-cold phosphate-buffered saline. Returned lavage fluid was pooled for each animal and centrifuged at 1000 ×g for 10 min at 4°C. The cell-free supernatants were harvested for total protein analysis using the BCA protein assay kit (Nanjing Jiancheng Bioengineering Institute, China).

### 2.7. Lung Water Content

Lung water content was used as an index to estimate the degree of pulmonary edema. After the animal was sacrificed, the lungs were excised en bloc, blot dried, and placed on preweighed glass plates. The wet weight of the tissue was registered immediately. Then the tissue was placed in an incubator at 80°C for 72 h to obtain a constant weight. After the dry weight of the tissue was registered, the water content of the tissue was calculated according to the formula: lung water content (%) = (wet weight − dry weight)/dry weight × 100% [9].

### 2.8. Histopathologic Analysis

The lungs were harvested at 10 h after LPS administration and fixed with an intratracheal instillation of 1 ml buffered formalin (10%, pH 7.2). The lobe was further fixed in 10% neutral buffered formalin for 48 h at 4°C. The tissues were embedded in paraffin wax. The sections approximately 5 *μ*m thick were stained with hematoxylin and eosin using a standard protocol and observed under a light microscope for histopathological changes such as alveolar septum lesion, inflammatory cell infiltration, and blood stasis.

### 2.9. Immunofluorescence Studies

Nuclear factor erythroid 2-related factor 2 (Nrf2) nuclear translocation in lung cells was determined by immunofluorescence technique described by Lindl and Sciutto [11]. The sections of the lung tissue prepared as mentioned above were deparaffinized and rehydrated through submersion in graded alcohols. Antigen retrieval was performed with 10 mM citrate buffer pH 6 for 5 min in a microwave oven. The antibody used for immunostaining the V5-tagged protein was anti-V5-FITC (Invitrogen), and for visualizing the endogenous protein, the cells were probed with Nrf2 antibody. The fluorescein isothiocyanate- (FITC-) conjugated anti-rabbit antibody was used as a secondary antibody. To visualize the nuclei, the cells were stained with DAPI. The fluorescent images were captured using appropriate filters in a Nikon inverted fluorescent microscope. The antibodies were achieved from Wuhan Servicebio Technology Co Ltd. (Wuhan, China).

### 2.10. Oxidative Stress Markers *In Vivo*

The lung samples were homogenized with KCl solution (0.154 mol/L) in ice-cold condition and centrifuged at 3000 ×g for 15 min at 4°C. The supernatants were immediately stored at *−*80°C until assayed for malondialdehyde (MDA), glutathione peroxidase (GSH-Px), and superoxide dismutase (SOD) using respective kits (Nanjing Jiancheng Bioengineering Institute, China) according to instruments provided by the manufacturer.

### 2.11. Western Blot Analysis

The lung tissue homogenate was centrifuged (12,000 ×g, 10 min, 4°C) and the supernatants were aspirated. Biochemical fractionation of the cells was done using the nuclear extract kit (Active Motif, USA) according to the manufacturer's instructions. Protein concentrations were determined by the Bradford method [12]. Proteins were loaded and transferred to a PVDF membrane (Millipore, USA). After being blocked, the membranes were incubated overnight at 4°C with anti-Nrf2 (1 : 1000, Millipore) antibodies. The membranes were then incubated for 1 h at room temperature with horseradish peroxidase- (HRP-) labeled goat anti-rabbit secondary antibody (1 : 4000, Vector, Burlingame, USA). The membranes were placed into a gel imaging system (Bio-Rad, ChemiDoc XRS, USA) and then exposed. The intensity of blots was quantified using the Quantity One analysis software (Bio-Rad, USA). Lamin B was used as an internal control.

### 2.12. Statistical Analysis

Data were expressed as mean ± standard deviation. All statistical analysis was performed with SPSS 17.0 software package (SPSS Inc., Chicago, USA). Statistically significant differences between groups were determined by ANOVA followed by Tukey's test. The results were considered statistically significant if *P* values were <0.05.

## 3. Results

### 3.1. Antioxidant Activity *In Vitro*

The results of the three different methods to evaluate radical scavenging activities indicated that SP has strong antioxidant activity in the same order of magnitude with that of the positive control trolox. The values of IC_50_ are shown in Table 2.

### 3.2. Body Weight Change Ratio

**Figure 1:**
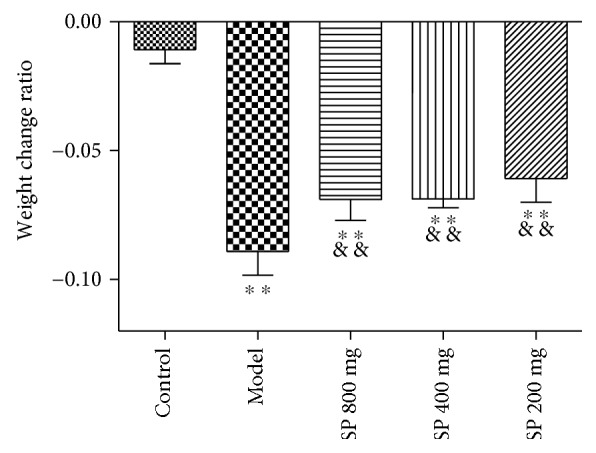
Body weight change ratios (*n* = 30). ^∗∗^*P* < 0.01 versus control; ^&&^*P* < 0.01 versus model.

LPS injection caused body weight loss in each treatment group. Ten hours after injection, the average weight of the model group was significantly reduced compared to that of the control group (*P* < 0.01). While SP provided protection to some extent, weight change ratios in all SP-treated groups were significantly decreased than that of the model group (*P* < 0.01), as shown in Figure 1. Low dose seemed to provide slightly better protection than medium and high dose (−0.061 ± 0.009, −0.069 ± 0.03, and −0.069 ± 0.008 for 200, 400, and 800 mg/kg dose, resp.). Whether the difference has practical significance is not clear yet.

### 3.3. Histopathologic Observations

**Figure 2:**
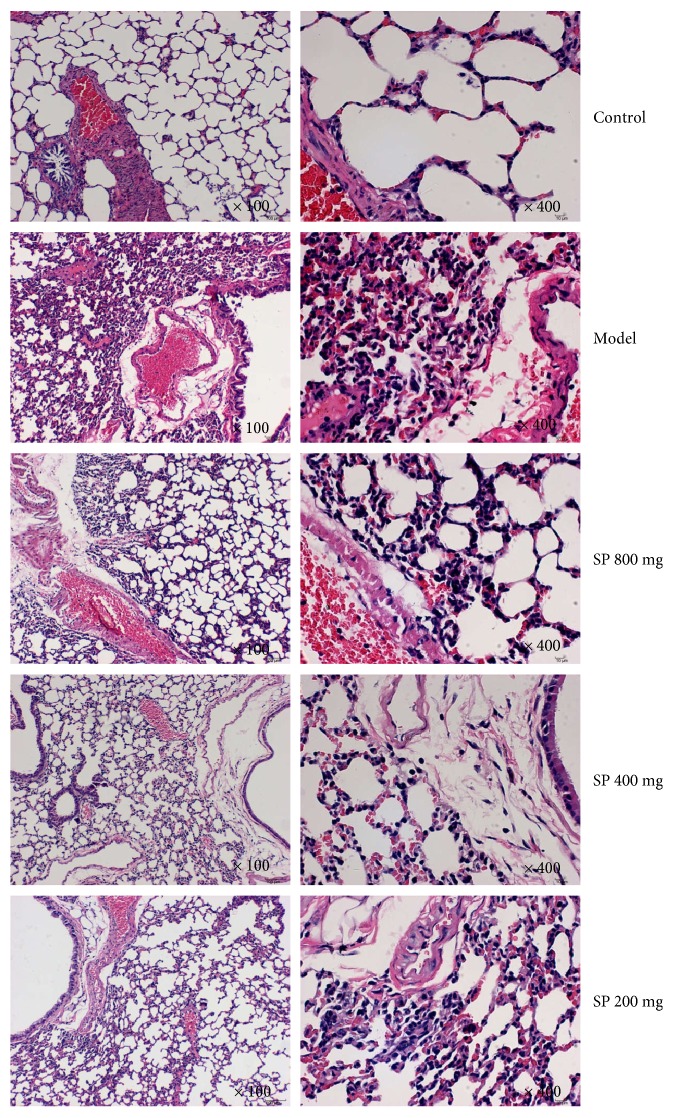
Effects of the seabuckthorn paste on the lung histopathological changes (hematoxylin-eosin stain, ×100 and ×400).

As shown in Figure 2, the lung tissues from the control group represented normal structure without histopathologic changes under a light microscope. In the LPS-induced ALI mice of the model group, the lungs stained with hematoxylin-eosin indicated widespread alveolar wall thickness caused by edema, severe hemorrhage in the alveolus, alveolus collapse, and obvious inflammatory cell infiltration. In the SP-pretreated groups, the histopathologic lesions were minor compared with those in the model group.

### 3.4. Lung Water Content

**Figure 3:**
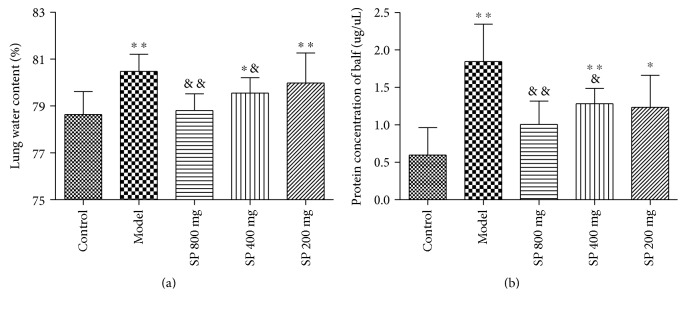
Effects of the seabuckthorn paste on the lung's transvascular leakage of fluid (a) and total proteins (b) (*n* = 10). ^∗^*P* < 0.05 or ^∗∗^*P* < 0.01 versus control; ^&^*P* < 0.05 or ^&&^*P* < 0.01 versus model.

The lung water content in normal control animals varied between 77.21% and 80.12% with a mean of 78.63 ± 0.98%. The mean value of 80.48 ± 0.73% in mice of the model group was significantly higher (*P* < 0.01) than that of the normal control group. The lung water contents in the SP 800 mg group and SP 400 mg group animals were both significantly lower (*P* < 0.01 and *P* < 0.05, 78.81 ± 0.72% and 79.55 ± 0.66% for 800 and 400 mg/kg dose, resp.) than that of the model group, while water content difference between the SP 200 mg/kg group and model group has no statistical significance. Animals in the 800 mg/kg SP group had substantial alleviation with no significant difference in water content comparing to that of the control group, as shown in Figure 3(a).

### 3.5. Total Protein Content in BALF

The mean BALF protein content in model animals was significantly higher (1.84 ± 0.50 mg/ml) (*P* < 0.01) as compared with the normal control values (0.60 ± 0.37 mg/ml). Pretreatment with SP provided remarkable protection against protein transvascular leakage, with BALF protein contents in high and medium SP dose groups significantly lower (*P* < 0.01 and *P* < 0.05, 1.00 ± 0.31and 1.28 ± 0.21 mg/ml for 800 and 400 mg/kg dose, resp.) than that of the model group, and the low SP dose group indicated a lower mean value yet without statistical significance (1.24 ± 0.43 mg/ml for 200 mg/kg dose), as shown in Figure 3(b). Notably, high dose of SP provided a strong protection that protein content in BALF in the 800 mg/kg group has no significant difference comparing to that of normal control animals.

### 3.6. Oxidative Stress Markers

LPS injection caused a significant increase (*P* < 0.01) (2.06 ± 0.36 nmol/mg) in the oxidative stress marker MDA levels in the lung homogenates than in the control group (1.22 ± 0.21 nmol/mg). The pretreatment of SP-attenuated MDA generation (1.66 ± 0.36, 1.83 ± 0.17, and 1.87 ± 0.14 nmol/mg for 800, 400, and 200 mg/kg dose, resp.) as compared with the model group and the difference between the 800 mg/kg group and model group have statistical significance (*P* < 0.01).

The levels of two antioxidant enzymes in the lung homogenates were significantly lower (*P* < 0.01) in the model group (4.37 ± 0.52 U/mg for SOD and 25.99 ± 2.81 *μ*mol/mg for GSH-Px) than that of the control group (6.01 ± 0.65 U/mg for SOD and 36.00 ± 1.92 *μ*mol/mg for GSH-Px). The pretreatment of animals with SP caused a marked increase in the level of SOD (5.61 ± 1.46, 5.43 ± 0.55, and 5.21 ± 0.66 U/mg for 800, 400, and 200 mg/kg dose, resp.) and GSH-Px (30.41 ± 4.40, 28.96 ± 3.97, and 31.34 ± 4.09 *μ*mol/mg for800, 400, and 200 mg/kg dose, resp.) as compared with the model group. Statistical significance is evident in 400 mg/kg dose for SOD (*P* < 0.01) and also in 800 and 200 mg/kg dose for GSH-Px (*P* < 0.01).

**Figure 4:**
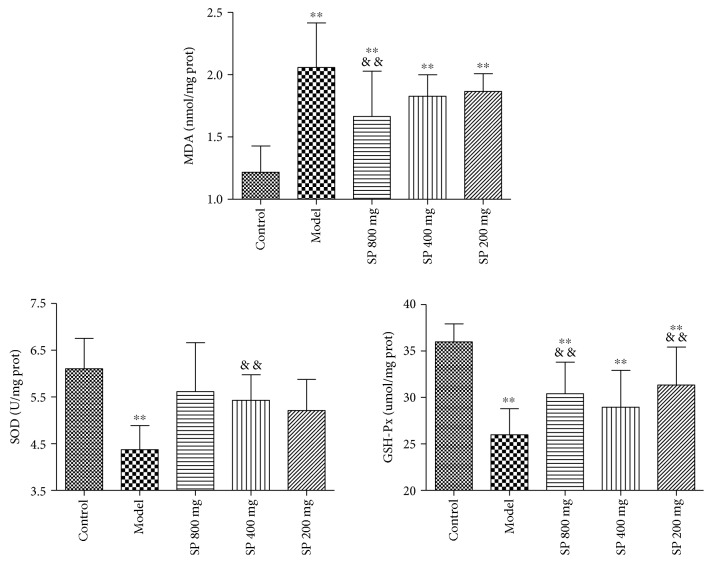
Effects of the seabuckthorn paste on the oxidative stress markers *in vivo* (*n* = 10). ^∗∗^*P* < 0.01 versus control; ^&&^*P* < 0.01 versus model.

These results are shown in Figure 4.

### 3.7. Lung Nrf2 Immunofluorescence Observations

**Figure 5:**
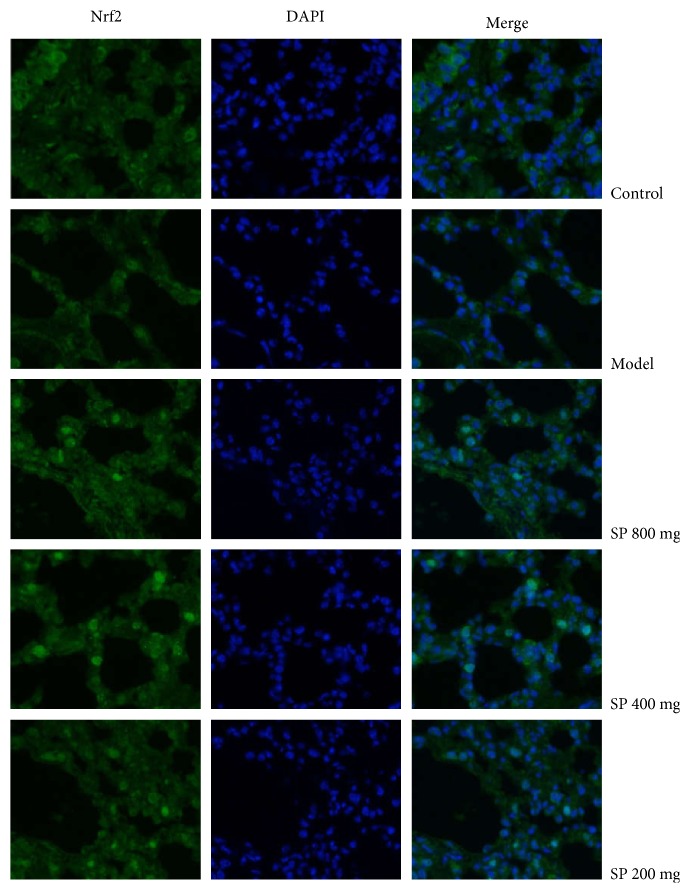
Effects of the seabuckthorn paste on Nrf2 nuclear translocation in lung cells (×400). Nrf2 localization was determined by immunofluorescence staining with anti-Nrf2 antibody followed by fluorescence-tagged secondary antibody. DAPI was used to visualize the nuclei in blue filter.

As evident from Figure 5, immunofluorescence results showed varying degrees of enrichment of the Nrf2 protein both in the nuclear fraction and in the cytoplasmic fraction in lung cells upon a different dose SP treatment, comparing with control and model groups. The increased Nrf2 protein in the nucleus suggested translocation of the Nrf2 protein into the nuclei, which is considered as the starting point of Nrf2 pathway activation.

### 3.8. Nrf2 Protein Expression in Lung Nuclei

**Figure 6:**
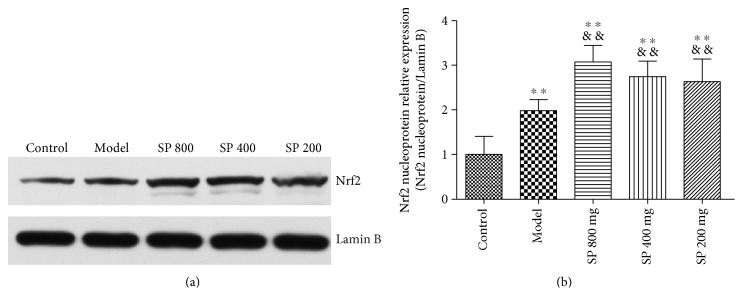
Effects of the seabuckthorn paste on the nuclear Nrf2 protein expression. Nuclear Nrf2 expression was detected by western blot (a) and the density of immunoreactive bands was analyzed (b). ^∗∗^*P* < 0.01 versus control; ^&&^*P* < 0.01 versus model.

As Figure 6 shows, SP treatment caused increased Nrf2 accumulation in the nuclear fraction. Nuclear Nrf2 level in the model group (0.30 ± 0.03) has a significant increase compared to that of the control group (0.15 ± 0.06) (*P* < 0.01), while more pronounced increases were observed in the SP groups of different dose (0.47 ± 0.06, 0.42 ± 0.05, and 0.40 ± 0.07 for 800, 400, and 200 mg/kg dose, resp.), all with statistical significance compared to that of the model group (*P* < 0.01 versus model group).

## 4. Discussion

In the present study, we have demonstrated that traditional Tibetan medicine seabuckthorn paste has strong radical scavenging activities on par with trolox. Seabuckthorn berries contain high amount of polyphenols especially flavonoids, which are partly responsible for its potent antioxidative capacity. Our results consisted with previous reports that seabuckthorn is a desirable natural antioxidant resource possessing a variety of functions that improve health, such as alleviating atherosclerosis, reversing hyperthyroidism, reducing inflammation, relieving gastric ulcers, and preventing liver cirrhosis [13, 14]. However, the harvest of seabuckthorn berries is seasonal, and matured berries are easily to decay. In order to access this valuable medicine whenever necessary, Tibetans developed a process to make these berries into a paste, which could be stored for a long time conveniently. The oxidation resistance evaluation results in this study proved that the preparation of SP retains high content of polyphenols/flavonoids and potent antioxidative activities, thus may have health benefits similar to seabuckthorn berries.

LPS exposure causes general toxic responses including body weight loss and anorexia [15, 16], which were effectively reversed by SP pretreatment in the present study. As for local lesions, microstructure changes were observed in the lungs under LPS challenge, with obvious alleviation in the mice of SP treatment groups as per expectation. These results demonstrated that administration of SP in mice provided remarkable protection against LPS-induced acute lung injury. Previous studies showed that LPS exposure increased airway epithelium barrier paracellular permeability, associated with elevated levels of leakage for fluids, proteins, and other vascular components [17–19]. And the increased leakage contributes to diseases such as acute respiratory distress syndrome and pulmonary edema. Pretreatment with SP caused a marked decline in transvascular fluid leakage into the lungs besides curtailing leakage of proteins into the alveoli. These results suggest that SP may help maintain alveolar arterial capillary membrane integrity, thereby blocking pathological progress towards lethal conditions.

We explored the protection mechanism of SP on LPS-induced acute lung lesion in a perspective of redox homeostasis. High-dose exposure of LPS can trigger toxic inflammatory reactions through reactive oxygen and nitrogen species, and excessive production of these radicals is implicated to damage biomembranes, thereby compromising cell integrity and function and resulting in increased pulmonary capillary permeability [20–22]. Hence, the oxidative stress marker (MDA) and antioxidant enzymes (SOD, GSH-Px) in the lung homogenates of different groups of animals were measured. The increase in level of MDA and decrease in levels of SOD and GSH-Px caused by LPS stimulation were reversed in animals of SP treatment groups, indicating that SP could provide a significant protection against LPS-induced oxidative damage.

Based on the positive antioxidant results *in vivo*, we investigated the involvement of the Nrf2 pathway under SP exposure. Nrf2 transcription factor is one of the most important antioxidant defense mechanisms that protect cells and tissues from various oxidative stresses. The accumulation of Nrf2 from the cytoplasm to nucleus is an essential signaling start for Nrf2 media regulation of antioxidant/detoxification enzymes [23, 24]. Immunofluorescence and western blot results in this study indicate that LPS stimulation increased the level of Nrf2 in the nuclei, as a stress response of the body defense system. And SP treatment resulted further elevation of Nrf2 accumulation in the nuclear fraction, confirming the occurrence of Nrf2 translocation and activation. Furthermore, dose-response relationships were observed in the assays. Therefore, the protective effect of SP on LPS-induced ALI is clearly associated with attenuation of oxidant stress, and the underlying mechanism through which SP exert antioxidant properties involves Nrf2 pathway activation.

In conclusion, we demonstrated that the seabuckthorn paste with relative high contents of polyphenols and flavonoids has potent antioxidant activities *in vitro* and *in vivo*. This traditional medicine can provide strong protection in mice against LPS-induced acute lung injury through maintaining redox homeostasis, and its mechanism involves Nrf2 nuclear translocation and activation.

## Figures and Tables

**Table 1:** Animal experimental protocol.

Mice	Analysis Items
Batch I (*n* = 50)	Body weight, lung water content
Batch II (*n* = 50)	Body weight, bronchoalveolar lavage fluid (BALF) analysis, histopathology, immunofluorescence
Batch III (*n* = 50)	Body weight, oxidative stress markers, western blot

Mice in each batch were divided into five groups: (i) normal control group, (ii) model group, (iii) high-dose SP group (200 mg/kg), (iv) medium-dose SP group (400 mg/kg), and (v) low-dose SP group (800 mg/kg) (*n* = 10 for each group).

**Table 2:** DPPH, ABTS, and hydroxyl radical scavenging activities and total polyphenols/flavonoids contents of the seabuckthorn paste.

Sample	IC_50_ (*μ*g/ml)^a^			Content (mg/g)	
	DPPH radical^b^	ABTS radical^b^	Hydroxyl radical^b^	Total polyphenols^b^	Total flavonoids^b^
SP	18.54 ± 0.78	9.75 ± 0.35	1.43 ± 0.10	191.5 ± 5.78	130.9 ± 4.25
Trolox^c^	5.39 ± 0.12	3.02 ± 0.11	1.07 ± 0.08	—	—

^a^IC_50_ (*μ*g/ml): the concentration at which 50% is inhibited. ^b^Each value is expressed as mean ± standard deviation (*n* = 3). ^c^Trolox as positive control.

## References

[1] Bakonyi T., Radak Z. (2004). High altitude and free radicals. *Journal of Sports Science & Medicine*.

[2] Dosek A., Ohno H., Acs Z., Taylor A. W., Radak Z. (2007). High altitude and oxidative stress. *Respiratory Physiology & Neurobiology*.

[3] Sato K., Kadiiska M. B., Ghio A. J. (2002). In vivo lipid-derived free radical formation by NADPH oxidase in acute lung injury induced by lipopolysaccharide: a model for ARDS. *The FASEB Journal*.

[4] Sabir S. M., Maqsood H., Imran H., Khan M. Q., Khaliq A. (2005). Elemental and nutritional analysis of sea buckthorn (Hippophae rhamnoides ssp. turkestanica) berries of Pakistani origin. *Journal of Medicinal Food*.

[5] Sawaf O. A., Clarner T., Fragoulis A. (2015). Nrf2 in health and disease: current and future clinical implications. *Clinical Science*.

[6] Kensler T. W., Wakabayashi N., Biswal S. (2007). Cell survival responses to environmental stresses via the Keap1-Nrf2-ARE pathway. *Annual Review of Pharmacology and Toxicology*.

[7] Debnath T., Park P. J., Debnath N. C., Samad N. B., Park H. W., Lim B. O. (2011). Antioxidant activity of Gardenia jasminoides Ellis fruit extracts. *Food Chemistry*.

[8] Hazra B., Biswas S., Mandal N. (2008). Antioxidant and free radical scavenging activity of Spondias pinnata. *BMC Complementary and Alternative Medicine*.

[9] Liu J., Wang C., Wang Z. Z., Zhang C., Lu S., Liu J. (2011). The antioxidant and free-radical scavenging activities of extract and fractions from corn silk (Zea mays L.) and related flavone glycosides. *Food Chemistry*.

[10] Chang C. C., Yang M. H., Wen H. M., Chern J. C. (2002). Estimation of total flavonoid content in propolis by two complementary colorimetric methods. *Journal of Food and Drug Analysis*.

[11] Lindl K. A., Sciutto K. L. (2008). Examining the endogenous antioxidant response through immunofluorescent analysis of Nrf2 in tissue. *Methods in Molecular Biology*.

[12] Bradford M. M. (1976). A rapid and sensitive method for the quantitation of microgram quantities of protein utilizing the principle of protein-dye binding. *Analytical Biochemistry*.

[13] Yang B., Kallio H. (2002). Composition and physiological effects of seabuckthorn (Hippophae) lipids. *Trends in Food Science & Technology*.

[14] Sabir S. M., Maqsood H., Imran H., Khan M. Q., Khaliq A. (2005). Elemental and nutritional analysis of sea buckthorn (Hippophae rhamnoides ssp. turkestanica) berries of Pakistani origin. *Journal of Medicinal Food*.

[15] Cerami A., Ikeda Y., Trang N. L. (1985). Weight loss associated with an endotoxin-induced mediator from peritoneal macrophages: the role of cachectin (tumor necrosis factor). *Immunology Letters*.

[16] Lee K., Ewing J. F., Sluss P. M. (1989). Effect of bacterial lipopolysaccharide on growth of murine bladder cancer, MBT-2. *Urological Research*.

[17] Eutamene H., Theodorou V., Schmidlin F. (2005). LPS-induced lung inflammation is linked to increased epithelial permeability: role of MLCK. *European Respiratory Journal*.

[18] Yang B., Li X. P., Ni Y. F. (2016). Protective effect of isorhamnetin on lipopolysaccharide induced acute lung injury in mice. *Inflammation*.

[19] Zhang H., Sun G. Y. (2005). LPS induces permeability injury in lung microvascular endothelium via AT_1_ receptor. *Archives of Biochemistry and Biophysics*.

[20] Kallapura G., Pumford N. R., Velasco X. H., Hargisand B., Tellez G. (2014). Mechanisms involved in lipopolysaccharide derived ROS and RNS oxidative stress and septic shock. *Journal of Microbiology Research and Reviews*.

[21] Vanita G., Asheesh G., Shalini S., Harish M. D., Grover S. K., Ratan K. (2005). Anti-stress and adaptogenic activity of L-arginine supplementation. *Evidence-Based Complementary and Alternative Medicine*.

[22] Herget J., Wilhelm J., Novotna J. (2000). A possible role of the oxidant tissue injury in the development of hypoxic pulmonary hypertension. *Physiological Research*.

[23] Sahin K., Orhan C., Tuzcu Z., Tuzcu M., Sahin N. (2012). Curcumin ameloriates heat stress via inhibition of oxidative stress and modulation of Nrf2/HO-1 pathway in quail. *Food and Chemical Toxicology*.

[24] Kaspar J. W., Niture S. K., Jaiswal A. K. (2009). Nrf2:INrf2 (Keap1) signaling in oxidative stress. *Free Radical Biology and Medicine*.

